# A survey to assess the extent of public-private mix DOTS in the management of tuberculosis in Zambia

**DOI:** 10.4102/phcfm.v7i1.692

**Published:** 2015-03-27

**Authors:** Gershom Chongwe, Nathan Kapata, Mwendaweli Maboshe, Charles Michelo, Olusegun Babaniyi

**Affiliations:** 1 University of Zambia, School of Medicine, Department of Public Health, Zambia; 2 Ministry of Health, National TB/Leprosy Control Program, Zambia; 3 World Health Organization Country Office, Zambia

## Abstract

**Background:**

Involving all relevant healthcare providers in tuberculosis (TB) management through public-private mix (PPM) approaches is a vital element in the World Health Organization's (WHO) Stop TB Strategy. The control of TB in Zambia is mainly done in the public health sector, despite the high overall incidence rates.

**Aim:**

We conducted a survey to determine the extent of private-sector capacity, participation, practices and adherence to national guidelines in the control of TB.

**Setting:**

This survey was done in the year 2012 in 157 facilities in three provinces of Zambia where approximately 85% of the country's private health facilities are found.

**Methods:**

We used a structured questionnaire to interview the heads of private health facilities to assess the participation of the private health sector in TB diagnosis, management and prevention activities.

**Results:**

Out of 157 facilities surveyed, 40.5% were from the Copperbelt, 4.4% from Central province and 55.1% from Lusaka province. Only 23.8% of the facilities were able to provide full diagnosis and management of TB patients. Although 47.4% of the facilities reported that they do notify their cases to the National TB control programme, the majority (62.7%) of these facilities did not show evidence of notifications.

**Conclusion:**

Our results show that the majority of the facilities that diagnose and manage TB in the private sector do not report their TB activities to the National TB Control Programme (NTP). There is a need for the NTP to improve collaboration with the private sector with respect to TB control activities and PPM for Directly Observed Treatment, Short Course (DOTS).

## Abstrait

**Une étude pour évaluer l'étendue des DOTS combinés publics-privés dans la gestion de la tuberculose en Zambie.**

**Contexte:**

La participation de tous les professionnels de santé concernés dans la gestion de la tuberculose (TB) par une approche combinée publique-privée (PPM) est un élément capital de la Stratégie « Halte à la Tuberculose (TB) » de l'Organisation mondiale de la Santé (OMS). Le contrôle de la tuberculose en Zambie se fait principalement dans le secteur de la santé publique, malgré le taux d'incidence global élevé.

**Objectif:**

Nous avons mené une enquête pour déterminer l'importance de la capacité, participation, pratiques et adhésion du secteur privé aux directives nationales pour le contrôle de la tuberculose.

**Cadre:**

Cette enquête a été faite en 2012 dans 157 établissements de santé dans trois provinces de la Zambie où se trouvent environ 85% des services de santé privés du pays.

**Méthodes:**

Nous avons utilisé un questionnaire structuré pour interviewer les directeurs des établissements sanitaires privés afin d'évaluer la participation du secteur de santé privé dans le diagnostic, la gestion et la prévention de la tuberculose.

**Résultats:**

Sur les 157 établissements examinés, 40.5% provenaient de la Copperbelt, 4.4% de la Province Centrale et 55.1% de la province de Lusaka. Seuls 23.8% des établissements pouvaient faire un diagnostic complet et gérer les tuberculeux. Bien que 47.4% des établissements aient déclaré qu'ils avaient signalé leurs patients au programme national de contrôle de la tuberculose, la majorité (62.7%) de ces établissements n'a pas pu donner de preuve de notifications.

**Conclusion:**

Nos résultats montrent que la majorité des établissements qui diagnostiquent et gèrent la tuberculose dans le secteur privé ne déclarent pas leurs activités concernant la tuberculose au programme de contrôle national de la tuberculose (NTP). Il faut que le NTP améliore sa collaboration avec le secteur privé par rapport aux activités de contrôle de la tuberculose et le PPM pour le Traitement de brève Durée sous Surveillance directe (DOTS).

## Introduction

Involving all relevant healthcare providers in tuberculosis (TB) care and control through public–private mix (PPM) approaches is a vital element in the World Health Organization's (WHO) Stop TB Strategy. PPM refers to the activities that link all healthcare facilities within the private and public sectors to national TB programmes for the expansion of Directly Observed Treatment, Short Course (DOTS) activities.^1^ The approach is one of the tools employed in the implementation of the International Standards for TB Care (ISTC) in order to achieve national as well as global TB targets. The use of private health providers has been shown to increase notification rates of smear-positive pulmonary TB by between 10% and 60%.^2^

The WHO estimates that there were 9 million new cases of TB in 2011, with 1.4 million TB deaths worldwide.^3^ Zambia is amongst the WHO's African Region TB high-burden countries. It is estimated that in 2012, there were 40 726 new TB case notifications (including relapse cases in Zambia), or 289/100 000 population. In the southern African region, only South Africa, Swaziland, Lesotho and Botswana notified more cases per 100 000 population compared with Zambia in the year 2012.^4^

Increasing private-sector participation in TB control services has been shown to be a cost-effective way of improving care as well as notification rates of TB.^5,6,7^ The improved notification rates are generally seen following greater collaboration between the National TB Control Programme (NTP) and the private sector;^8,9^ however, other studies have suggested that consulting the private sector after the commencement of TB-related signs and symptoms may result in diagnostic and treatment delay.^10,11,12,13^ The extent of the PPM for TB care and control in Zambia is not known.

Whilst the scale up of TB/HIV collaborative activities in Zambia has increased over the past decade, challenges still remain in getting service delivery points, including private healthcare providers, to adhere to recommended standards.^14^ Private healthcare facilities are expected to take part in TB and HIV control activities such as diagnosis of cases, treatment and notification of cases. Information on the capacity to diagnose TB in the private health facilities, notification of cases to the authorities, the outcomes of treatment, as well as adherence to national treatment guidelines, is not available.

The scaling up of TB and/or HIV collaborative activities is one of the strategies of the Global Plan to Stop TB. According to the WHO world TB report for 2012, Zambia was amongst the countries that reported HIV testing rates of more than 85% in the year 2011.^3^ With the prevalence of HIV amongst TB patients in Zambia estimated at 65% – 68%,^3,15^ testing all TB patients for HIV is vital as it ensures that patients receive the appropriate care, which may include prophylactic Cotrimoxazole and/or antiretroviral therapy.

### Aims and objectives

We carried out a survey to determine the extent of private-sector capacity and participation in the provision of tuberculosis control services in Zambia, as well as to assess the private provider practices and adherence to national guidelines.

## Research methods and design

### Study design and setting

A cross-sectional survey was done in Lusaka, Central and Copperbelt provinces, where about 85% of all private healthcare facilities in Zambia are found. There were two survey teams, one for Lusaka and another for the Central and Copperbelt provinces. Team members comprised public health-sector workers serving as TB focal point persons. The team members were trained on how to use a validated semi-structured questionnaire (see Annexure 1) which was used to collect information from the heads of the private health facilities. The questionnaire included questions on: staff availability; capacity to diagnose TB using chest X-ray, smear microscopy or culture methods; and ability to manage a case of TB by providing drugs and other services. The data were collected between September and October 2012 as part of operations research on behalf of the NTP.

### Sampling strategy

A list of health facilities obtained from the Health Professions Council of Zambia, the body responsible for the licensing and regulation of all private health facilities in the country, was used as a sampling frame. Out of a total of 423 facilities on the list, 243 were in Lusaka and 180 from the Copperbelt province. We included facilities that offered general medical services regardless of their size.

Facilities such as dental clinics, optician clinics as well as clinics offering only reproductive health services were excluded. We also excluded mine underground clinics as well as first aid clinics within the mining plants and in workplaces. A number of facilities could not be located, mainly because they had closed or had moved to another area. After this process, the survey was conducted in the remaining 157 facilities, 87 of them in Lusaka, 63 from the Copperbelt province and 7 in the Central province.

### Data analysis

The data was entered into a questionnaire in Epidata statistical software, then analysed using STATA 11.2 software (StataCorp 2011). Proportions were expressed as percentages with corresponding 95% confidence intervals.

### Ethical considerations

A waiver was obtained from the University of Zambia Biomedical Research Ethics Committee to use these results arising from an operations research study by the National TB Control Programme.

## Results

Out of 157 facilities, 63 (40.5%) facilities were from the Copperbelt province, 7 (4.4%) from Central province and 87 (55.1%) from Lusaka province. Eighty-nine per cent (*n* = 139) of the facilities were privately owned, 5.1% (*n* = 8) owned by faith-based institutions and 6.4% (*n* = 10) owned by quasi-government or parastatal (or semi-autonomous, government-supported) institutions. More than 90% (*n* = 142) of the facilities were manned by at least one doctor. However, the number of doctors working in the facilities ranged from zero in 9.5% (*n* = 15) of the facilities to 17 doctors found in one of the largest facilities. Furthermore, 38.6% (*n* = 140) of the facilities had at least one inpatient bed for admission of patients.

### Management of tuberculosis cases

Only 23.8% (*n* = 151 [95% CI 17.0, 30.7]) of the facilities are able to fully diagnose and manage TB patients by conducting sputum smears or culture, chest X-ray examinations, as well as providing anti-TB drugs. Over half of the facilities (78 of 151; 51.7% [95% CI 43.6, 59.7]) reported that they routinely referred their suspected TB patients to the public sector whenever a case of TB is suspected, whereas 24.5% (*n* = 37 [95% CI 17.6, 31.4]) referred patients only after making the diagnosis. All the facilities that manage TB patients reported that they were able to provide TB drugs to their patients. Of these, 27.3% (*n* = 12) said they obtained their drugs from their own suppliers, whereas 72.7% (*n* = 32) of the providers obtained their drugs from the NTP through the respective district medical offices.

### Notification of tuberculosis

Although 47.4% (*n* = 36 [95% CI 34.6, 57.5]) of the facilities reported that they do notify their cases to the NTP, 62.7% (*n* = 47 [95% CI 51.5, 73.9]) of these could not show evidence of this when asked to produce a treatment register.

### Treatment guidelines

Only 36% (*n* = 48 [95% CI 27.8, 44.2]) of the respondents said they had been trained in the use of the national treatment guidelines, 79.2% (*n* = 38) of whom were trained through their respective district medical offices. Other training was provided by non-governmental organisations (NGOs). A total of 32 (66.7%) were trained using the latest guidelines introduced in 2007, whilst 16 (33.3%) were trained before the latest guidelines. Sixty-seven per cent (*n* = 63 [95% CI 57.3, 76.7]) of the respondents reported following the treatment guidelines when managing their patients.

### Capacity to diagnose tuberculosis

We investigated the capacity for diagnosis of TB in the private health sector. Of all the facilities visited, only 42 (26.9%) had capacity to examine sputum smears (95% CI 20.1, 34.6). When disaggregated according to the type of facility, 5 of the 10 (50%) parastatal or government supported facilities had such capacity, compared with only 36 of the 139 (30%) private facilities and one (12.5%) of the faith-based institutions. Further, only 9 facilities (6.7% [95% CI 2.6, 10.7]) had the capacity to culture sputum for mycobacteria. X-ray facilities were available in 21.1% (*n* = 30 [95% CI 13.4, 26.3]) of the facilities, with parastatal and private facilities having similar capacity at 20.0% (*n* = 2) and 21.7% (*n* = 35) respectively. This is shown in Table 1. A few of the facilities^3^ reported using rapid tests for TB diagnosis.

**TABLE 1:** Availability of diagnostic services in private health facilities.

Type of facility	Perform sputum smear *n* (%)	Perform sputum culture*n* (%)	Chest X-ray *n* (%)	Provide tuberculosis drugs *n* (%)
Parastatal (*N* = 10)	5 (50.0)	1 (11.1)	2 (20.0)	6 (60.0)
Faith-based facility (*N* = 8)	1 (12.5)	0 (0.0)	1 (12.5)	3 (50.0)
Private (*N* = 139)	36 (30.0)	8 (6.7)	35 (21.7)	35 (29.7)
**Total (*N* = 156)**	**42 (26.9)**	**9 (6.7)**	**30 (21.1)**	**44 (32.8)**

### Tuberculosis and/or HIV collaborative services

Cotrimoxazole prophylaxis is provided to 58.8% (*n* = 77 [95% CI 49.8, 67.3]) of HIV patients. When asked whether they test their TB patients for HIV, 83 (66.4%) of the facilities replied in the affirmative (95% CI 57.4.0, 74.6). On the other hand, only 57.3% (*n* = 67 [95% CI 47.8, 66.4]) of the respondents reported screening their HIV patients for TB. Over 43% (*n* = 57 [95% CI 34.9, 52.4]) of the facilities reported the ability to provide antiretroviral (ARV) drugs to their patients. The proportion of facilities providing ARVs was higher in the parastatal health facilities than in those owned by faith-based institutions or private individuals, as is shown in Table 2.

**TABLE 2:** Availability of tuberculosis/HIV collaborative services in private health facilities.

Type of facility	Provide Cotrimoxazole prophylaxis *n* (%)	Screen HIV patients for tuberculosis *n* (%)	Test tuberculosis patients for HIV *n* (%)	Provide anti-HIV drugs *n* (%)
Parastatal (*N* = 10)	7 (70.0)	7 (70.0)	7 (70.0)	7 (70.0)
Faith-based facility (*N* = 8)	4 (57.1)	3 (60.0)	4 (80.0)	3 (42.9)
Private (*N* = 139)	66 (57.9)	57 (51.8)	72 (70.6)	47 (41.2)
**Total (*N* = 156)**	**77 (58.8)**	**67 (57.3)**	**83 (66.4)**	**57 (43.5)**

## Discussion

### Key findings

Our results have shown that only a quarter of the private health facilities have a self-reported capacity to diagnose and manage TB cases in Zambia. Over three quarters of the facilities did not have capacity to either diagnose or manage tuberculosis cases and referred their patients to the public sector. Some studies have suggested that consulting the private sector after the commencement of TB-related signs and symptoms may result in diagnostic and treatment delay.^10,11,12^ Ensuring that private providers have the necessary skills to diagnose and manage TB patients will improve notifications, possibly improving treatment outcomes and preventing the development of multi-drug resistant TB.

The need to notify TB cases is essential for TB control. The lack of notification by almost two-thirds of the private-sector providers, despite this being a legal requirement, is an opportunity for the NTP and other partners to engage private practitioners through the respective district medical offices on the need to notify TB cases. A well-established PPM strategy has the potential to improve case notifications by between 10% – 60%.^2^ An analysis of different PPM models for TB control in Pakistan in order to estimate the contribution of the various private providers to TB case notification showed that the NGO model made the greatest contribution to case notification (58.3%), followed by the hospital-based model at 18.9%.^16^

### Discussion of key findings

The scaling up of TB and/or HIV collaborative activities is one of the strategies of the Global Plan to Stop TB. According to the WHO world TB report for 2012, Zambia was amongst the countries that reported HIV testing rates of more than 85% in 2011.^3^ These data are collected mainly from the public health sector. Our study has shown that only 70.5% of TB patients are tested for HIV in the private sector. With the prevalence of HIV amongst TB patients in Zambia estimated at 65% – 68%,^3,15^ testing all TB patients for HIV is vital as it ensures that patients receive the appropriate care, which may include prophylactic Cotrimoxazole and/or anti-retroviral therapy.

### Strengths and limitations

This is the first report on PPM DOTs in Zambia. Although many patients in Zambia seek medical attention from a myriad of practitioners, ranging from traditional healers to formal western-oriented medical practitioners, our study did not investigate the role played by traditional practitioners. It is not known what proportion of Zambians with TB seek medical attention from traditional practitioners, nor do we know what their practices are with regard to patients presenting with symptoms suggestive of TB. There is therefore a need to investigate the role of traditional health practitioners in TB care in Zambia, as it has been shown that up to 30% – 40% of TB patients would have been seen by a traditional healer prior to a TB diagnosis in other places.^17,18^ The results could also have been affected by the differences in the size and capabilities of the facilities that were included in the survey as well as the presence of NTP staff amongst the data collectors.

### Recommendations

There is need for the NTP to improve collaboration with the private sector with respect to TB control activities and PPM, especially seeing that the majority of the facilities do not report their cases to the national programme.

There is also a need to improve training of the private health practitioners in the latest TB treatment guidelines. All necessary efforts must be made by the NTP and stakeholders to ensure that knowledge transfer of the most effective, latest and evidence-based treatment guidelines are disseminated widely to the private sector if TB control is to be achieved. District medical offices must improve collaboration and oversight roles in order to improve notification of TB cases by the private sector. TB and/or HIV collaborative activities in the private sector need to be enhanced as part of the PPM activities and the NTP must make efforts to ensure that every practitioner adheres to the ISTC.^19^

## Conclusion

Our results have shown that private sector participation in TB control and care is suboptimal. Whilst all the private facilities participate in the national TB programme either by diagnosing patients or referring them to other facilities for further management, the majority of the facilities did not show evidence of notification of cases to the NTP.
